# Impact of the COVID-19 pandemic on patients with pre-existing anxiety disorders attending secondary care

**DOI:** 10.1017/ipm.2020.75

**Published:** 2020-06-08

**Authors:** R. Plunkett, S. Costello, M. McGovern, C. McDonald, B Hallahan

**Affiliations:** 1 Galway-Roscommon Mental Health Services, University Hospital Galway, Galway, Ireland; 2 School of Medicine, National University of Ireland Galway, Galway, Ireland

**Keywords:** Anxiety disorders, COVID-19, obsessive compulsive disorder

## Abstract

**Objectives:**

To examine the psychological and social impact of the COVID-19 pandemic on patients with established anxiety disorders during a period of stringent mandated social restrictions.

**Methods:**

Semi-structured interviews were conducted with 30 individuals attending the Galway-Roscommon Mental Health Services with an International Classification of Diseases diagnosis of an anxiety disorder to determine the impact of the COVID-19 restrictions on anxiety and mood symptoms, social and occupational functioning and quality of life.

**Results:**

Twelve (40.0%) participants described COVID-19 restrictions as having a deleterious impact on their anxiety symptoms. Likert scale measurements noted that the greatest impact of COVID-19 related to social functioning (mean = 4.5, SD = 2.9), with a modest deleterious effect on anxiety symptoms noted (mean = 3.8, SD = 2.9). Clinician rated data noted that 8 (26.7%) participants had disimproved and 14 (46.7%) participants had improved since their previous clinical review, prior to commencement of COVID-19 restrictions. Conditions associated with no ‘trigger’, such as generalised anxiety disorder, demonstrated a non-significant increase in anxiety symptoms compared to conditions with a ‘trigger’, such as obsessive compulsive disorder. Psychiatric or physical comorbidity did not substantially impact on symptomatology secondary to COVID-19 mandated restrictions.

**Conclusions:**

The psychological and social impact of COVID-19 restrictions on individuals with pre-existing anxiety disorders has been modest with only minimal increases in symptomatology or social impairment noted.

## Introduction

The novel Coronavirus SarsCo-V2 was discovered in Wuhan in December 2019, following a cluster of patients who presented with severe viral pneumonia (Chan *et al.* 2020). The disease associated with COVID-19 spread rapidly in China with over 75,000 cases reported by 20 February 2020. Within weeks, COVID-19 had spread widely and rapidly throughout the world with a global pandemic declared by the World Health Organisation (WHO) on 11 March 2020. The first case of COVID-19 was documented in the Republic of Ireland on 27 February 2020. As of the 12 May 2020, there were 4,275,588 cases and 287,670 deaths attributed to COVID-19 (www.worldometers.info) worldwide, with 23,242 confirmed cases and 1,488 deaths attributed to COVID-19 in Ireland (www.gov.ie/en/news/7e0924-latest-updates-on-covid-19-coronavirus). These figures likely underestimate the true case numbers given that many individuals are asymptomatic, have a relatively benign illness course requiring minimal or no medical management or engage in preventative measures despite symptomatology without testing for COVID-19.

The potentially devastating medical, economic, social, cultural and psychological impact of a viral pandemic is well established (Nicola *et al*. 2020). A commission under the National Academy of Medicine in the United States of America predicted 4 years ago that a pandemic on the scale of the Spanish Flu pandemic of 1918–1921 could result in a $6 trillion loss to world economies (Sands, Mundaca-Shah & Dzau, 2016), with significant concern and growing evidence of a developing worldwide recession and financial collapse affecting multiple business sectors (Nicola *et al*. 2020). The social effects of a pandemic may include disruption of daily routine, social isolation including separation from family and friends, potential shortages of food and medicine, increased risks of exposure to domestic abuse for individuals in lockdown and online gaming (Taylor, 2019; www.gov.uk/guidance/domestic-abuse-how-to-get-help). Increasing debate and discussion in medical literature and in social media has surrounded the potential adverse psychological or psychiatric sequelae relating to COVID-19 (Cullen *et al.* 2020; Xiang *et al.* 2020). Previous viral pandemics have been associated with increased psychological distress (WHO “Outbreak Communication Guidelines”, 2005). Perspective pieces (Kelly, 2020) and some initial research studies note an increase in psychiatric pathology, including in mood and anxiety symptoms, in individuals with no prior mental disorder subsequent to mandated governmental restrictions secondary to COVID-19 (Wang *et al.* 2020). However, to date, there have been no published studies examining the impact of the COVID-19 pandemic on individuals with pre-existing diagnosed mental disorders who are attending secondary mental health services. Anxiety disorders are particularly important to explore given the potential adverse impact on anxiety of COVID-19 (Wang *et al.* 2020). Obsessive compulsive disorder (OCD) is particularly worthy of exploration given current strong recommendations on hand washing, as this anxiety disorder is marked by the presence of recurrent obsessional thoughts often encompassing contamination, and associated compulsive rituals incorporating repetitive hand washing, cleaning and/or undertaking disproportionate measures to reduce exposure to perceived sources of contamination (Murphy *et al*. 2010; Rasmussen & Eisen, 1992).

Consequently, in this study, we wanted to assess the psychological and social impact of COVID-19 including its associated mandated social restrictions on individuals with diagnosed anxiety disorders attending a general adult mental health service. We hypothesised that participants with anxiety disorders would have increased symptomatology and impaired social functioning and wondered whether this would be more prevalent in individuals with anxiety disorders associated with particular triggers, such as OCD.

## Methods

### Participants

Patients attending a single sector-based adult community mental health team for the management of an anxiety disorder (n = 54) were invited to participate in this study by letter and subsequently phoned by researchers (RP, SC, MMcG, BH) to provide clarification of the purpose of and procedure associated with this study. Patients with anxiety disorders comprised 14.4% of patients attending the community mental health team (mean age 44.9 years (SD = 15.1). Anxiety disorders consisted of those related to triggering events denoted as ‘trigger’ disorders and included OCD, social phobia and agoraphobia and those predominantly unrelated to a trigger event denoted as ‘non-trigger’ disorders and included GAD, panic disorder and mixed anxiety and depressive disorder. Clinical diagnoses were based on International Classification of Diseases (ICD-10) diagnostic criteria (WHO, 2004) with all diagnoses reviewed and confirmed by the consultant psychiatrist responsible for their care prior to study participation. Inclusion criteria for the study required patients to have one of these listed anxiety disorders, be over 18 years of age and have the capacity to provide written informed consent for study participation. Participants were excluded if they fulfilled criteria for an intellectual disability (intelligence quotient < 70) or had a confirmed diagnosis of dementia. Research interviews were undertaken by psychiatrists with several years of clinical practice (RP, SC, MMcG, BH) with training in study procedures provided by the principal investigator (BH). All responses were anonymised, and all data stored securely and handled in accordance with the Data Protection Act, 2018.

### Procedure

For individuals providing written informed consent (n=30), clinical case notes were reviewed to attain basic demographic and clinical data. Demographic data included age, gender, marital, domiciliary and employment or vocational status. Clinical data included psychiatric diagnosis and prescribed psychotropic medications including dose of medications, alcohol, tobacco and psycho-active substance use.

### Assessments

A semi-structured interview was conducted either in person or by telephone with participants who provided written informed consent. Interviews were conducted by telephone in the majority of cases (n=24) in-line with governmental and health service policy that ‘all non-essential surgery, health procedures and other non-essential health services [should] be postponed’. https://www.gov.ie/en/speech/f27026-speech-of-an-taoiseach-leo-varadkar-td-government-buildings-27-march/. In cases where the participant was already attending a pre-arranged outpatient appointment, interviews were conducted in person (n=6).

All data collection was carried out between 20 April and 7 May 2020, approximately 5–7 weeks after governmental mandated social restrictions (referred to colloquially as ‘lockdown’) had commenced. Demographic and clinical variables data attained from clinical note review were supplemented where required by data attained from clinical interviews, with additional information pertaining to physical health status including COVID-19 diagnosis and testing status, current domiciliary status and effect of COVID-19 on the participants’ employment or vocational status or site of employment.

Categorical data pertaining to the effect of COVID-19 on participants’ mental health status overall and severity of anxiety symptoms (better, no change, worse) were attained. Participants’ subjective experience of the impact of COVID-19 pandemic was additionally measured utilising Likert scales (0–10) to measure: (1) anxiety symptoms, (2) mood symptoms, (3) social functioning, (4) occupational functioning and (5) quality of life, with 0 indicating no adverse impact and 10 indicating a very severe impact due to restrictions imposed because of the COVID-19 pandemic.

Established psychometric instruments with known high reliability and validity indices were utilised to measure current symptomatology and include (1) Beck Anxiety Inventory (BAI, Steer *et al.* 1993), (2) Hamilton Anxiety Rating Scale (Ham-A, Hamilton, 1959), (3) Clinical Global Impression-Severity (CGI-S, Guy, 1976), (4) Global Assessment of Function (GAF, Hall, 1995) and (5) the Yale Brown Obsessive Compulsive Scale (Y-BOCS, Goodman *et al.* 1989) (for participants with a diagnosis of OCD only (n = 12)). The CGI-I scale was additionally utilised to compare participants’ current overall mental state to the last observation by their clinical team prior to the implementation of COVID-19 restrictions, typically 1–2 months prior to the introduction of these restrictions. Where uncertainty was present in relation to the previous clinical status of participants, the consultant psychiatrist or other senior members of the clinical team were contacted with patient consent to ensure accuracy of assessment.

### Statistical analysis

Statistical analysis was performed using the Statistical Package for Social Sciences (SPSS) 24.0 for Windows (SPSS Inc., IBM, New York, USA). Descriptive analyses (frequencies, percentages, means and standard deviation) on key demographic and clinical data were performed for both categorical and continuous variables, as appropriate. We utilised the Student’s t-test for parametric data and the Chi-square (*χ* ^2^) test for non-parametric data as appropriate. CGI-I data were combined due to the relatively small sample size into three categorical variables: improved (1–3), no change (4) or disimproved (5–7). Clinical data were compared between ‘trigger’ and ‘non-trigger’ disorders, between individuals with and without a diagnosis of OCD and between individuals with and without a diagnosed co-morbid mental health or physical health disorder. Data were examined to determine if normally distributed by visual inspection utilising histograms and by Q-Q plots and non-parametric testing of continuous data utilising the Mann–Whitney U test were additionally undertaken as appropriate. All statistical tests were two-sided, and the α-level for statistical significance was 0.05.

## Results

### Demographic and clinical data

Of the 54 participants initially invited to participate in this study, 12 were uncontactable and 12 declined to participate, resulting in a 55.6% overall response rate. There was no significant difference in terms of gender, age or anxiety disorder diagnosis between respondents and non-respondents. Data for the 30 study participants are presented in Table 1. Of note, 18 (60.0%) participants were female, the mean age of participants was 38.8 (SD = 12.8) years, 18 (60.0%) participants were residing with family members or a partner or spouse and prior to COVID-19 restrictions 10 (33.3%) participants were engaged in employment or third-level education. Of those employed, six individuals (66.7%) had their employment temporarily terminated, and the other three individuals (33.3%) had their site of work moved to their own residence due to COVID-19 restrictions. Seventeen participants (56.7%) fulfilled criteria for an anxiety disorders denoted as ‘trigger disorders’. The most common anxiety disorder was OCD (n = 2, 40.0%) followed by GAD (n = 10, 30%). Twenty-six (86.7%) participants were prescribed psychotropic medication with 13 (43.3%) participants prescribed a selective serotonin reuptake inhibitor, and 10 (30.0%) participants prescribed a serotonin and noradrenaline reuptake inhibitor. Twelve (40.0%) participants were prescribed more than one psychotropic medication. Thirteen (43.3%) participants had an additional diagnosed mental health disorder, with Emotionally Unstable Personality Disorder of Borderline type (n=5, 16.7%) the most common co-morbid mental health disorder. Nine participants (30.0%) had a diagnosed significant physical health disorder requiring ongoing medical management.

**Table 1.** Demographic and clinical data

Variable	n (%)
**Gender**	
Male	12 (40.0)
Female	18 (60.0)
**Marital Status**	
Single	18 (60.0)
Married/Civil Partnership	6 (20.0)
Separated/Divorced	6 (20.0)
**Employment**	
Unemployment	20 (66.7)
Employed	10 (33.3)
Lost employment (COVID-19)	6 (20.0)
In third-level education	1 (3.3)
**Anxiety Disorder**	
‘*Trigger’ anxiety disorders*	
Obsessive compulsive disorder	12 (40.0)
Social phobia	3 (10.0)
Agoraphobia	2 (6.7)
*‘Non-trigger’ anxiety disorders*	
Generalised anxiety disorder	9 (30.0)
Mixed anxiety and depression	3 (10.0)
Panic disorder	1 (3.3)
**Domiciliary Status**	
Parents	7 (23.3)
Partner/Spouse	7 (23.3)
Other family members	6 (20.0)
Housemates/Friends	3 (10.0)
Alone	7 (23.3)
**Substance Use**	
Alcohol*	15 (50.0)
Nicotine	4 (13.3)
Cannabis*	1 (3.3)
Other psycho-active substances	0 (0.0)
**Throat and Nasal Swab Test for COVID-19**	
Yes**	2 (6.7)
No	28 (93.3)
**Co-morbid Psychiatric Disorder**	
EUPD of Borderline Type	5 (16.7)
Schizophrenia	3 (10.0)
Anorexia nervosa	3 (10.0)
Other disorders***	2 (6.7)
**Co-morbid Physical Disorder**	
Diabetes mellitus	3 (10.0)
COAD/Asthma	3 (10.0)
Other physical health disorders****	3 (10.0)

COAD = chronic obstructive airway disease; EUPD = emotionally unstable personality disorder

* No participants fulfilled criteria for harmful use or dependence

** Both individuals tested negative for COVID-19

*** Includes autism spectrum disorder

**** Included neurological and musculoskeletal disorders

### Symptomatology

Low levels of anxiety symptoms were demonstrated as measured subjectively utilising the BAI (mean 12.5, SD = 12.0) and objectively utilising the HAM-A (mean 11.0, SD = 7.9). There was no difference in the level of anxiety symptoms between individuals with a ‘trigger’ or ‘non-trigger’ disorder (Table 2). Participants demonstrated on average a moderate impairment in functioning (GAF = 62.0, SD = 12.0) and 15 (50.0%) participants utilising the CGI-S were denoted as having a mental illness of mild severity, with the level of severity of illness more marked in individuals with a ‘trigger disorder’ (p = 0.03). For individuals with OCD, a mild-to-moderate level of symptomatology was demonstrated with a mean obsessional subscale score of 6.8 (SD = 3.2) and ritual subscale score of 7.6 (SD = 3.8) observed.

**Table 2.** ‘Trigger’ compared to ‘non-trigger’ anxiety disorders

Variable	Trigger Disorder (n = 17) n (%)	Non-Trigger Disorder (n = 13) n (%)	Statistics χ^2^, df, p
**COVID-19 testing**	1 (5.9)	1 (7.7)	0.04, 1, 0.51*
**CGI-S**			10.32, 5, 0.029*
Not currently ill	0 (0.0)	1 (7.7)	
Borderline illness	4 (23.5)	0 (0.0)	
Mild illness	5 (29.4)	10 (76.9)	
Moderate illness	6 (35.3)	2 (15.4)	
Marked illness	1 (5.9)	0 (0.0)	
Severe illness	1 (5.9)	0 (0.0)	
**Effect on Mental Health**			0.83, 1, 0.66*
Improvement	1 (5.9)	0 (0.0)	
No change	8 (47.1)	6 (46.2)	
Disimprovement	8 (47.1)	7 (53.8)	
**Effect on Anxiety Symptoms**			2.59, 2, 0.33*
Improvement	3 (17.6)	0 (0.0)	
No change	7 (41.2)	7 (53.8)	
Disimprovement	6 (46.2)	6 (46.2)	
**Clinician Rated Impression**			1.64, 2, 0.44*
Improvement	9 (52.9)	5 (38.5)	
No change	5 (29.4)	3 (23.1)	
Disimprovement	3 (17.6)	5 (38.5)	

	Mean (SD)	Mean (SD)	t, p
**Psychometric Data**			
BAI	10.70 (9.96)	14.92 (14.29)	0.56, 0.35
HARS	10.77 (6.96)	11.33 (9.43)	0.86, 0.85
GAF	59.41 (13.63)	65.38 (8.90)	0.22, 0.16
Y-BOCS (n = 12)	14.33 (6.16)	-	-
**Likert Scales Utilised**			
Anxiety	3.5 (2.8)	4.2 (3.2)	0.70, 0.49
Mood	3.4 (3.4)	3.2 (3.3)	0.10, 0.92
Social functioning	4.4 (3.1)	4.7 (2.9)	0.25, 0.80
Occupational functioning	2.9 (3.5)	3.2 (3.5)	0.60. 0.67
Quality of life	4.0 (2.7)	4.5 (2.7)	0.93, 0.59

CGI-I = Clinical Global Impression Improvement, CGI-S = Clinical Global Impression Severity, BAI = Beck Anxiety Inventory, HAMA-A = Hamilton Anxiety Rating Scale, GAF = Global Assessment of Function, Y-BOCS = Yale-Brown Obsessive Compulsive Scale

* Fisher’s Exact Test utilised

Clinician Rated Impression = CGI-I modified version

### Clinical effects of COVID-19

**Fig. 1.**
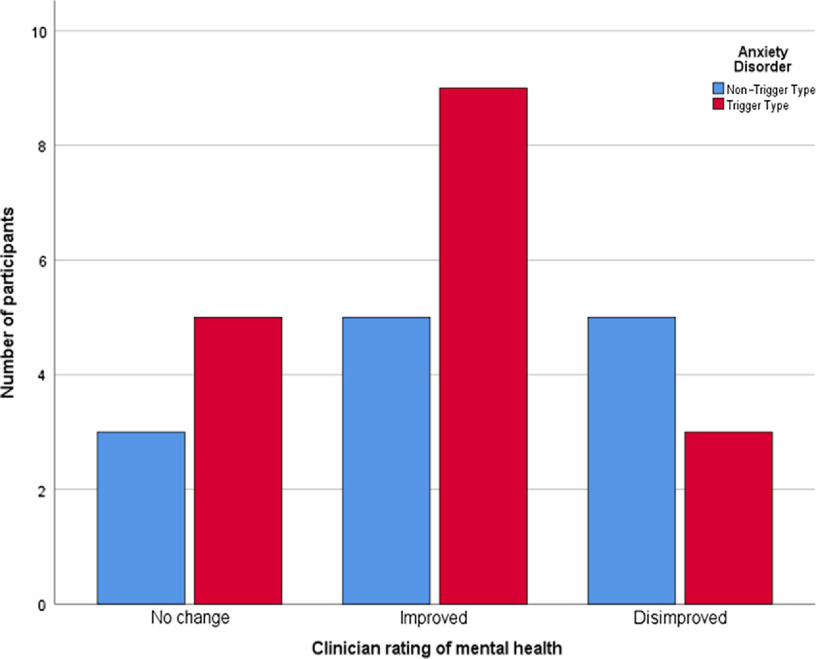
Clinical impression and ‘trigger’ v. ‘non-trigger’ anxiety disorders.

Fifteen (50.0%) participants described a deleterious effect of the COVID-19 pandemic on their mental health with 12 (40.0%) describing a deleterious effect pertaining to their levels of anxiety. The greatest impact of COVID-19 was on social functioning (mean = 4.5, SD = 2.9), followed by quality of life (mean = 4.2, SD = 2.6), with a modest deleterious effect on anxiety symptoms noted utilising Likert scale measurements (mean = 3.8, SD = 2.9). COVID-19 restrictions were associated with higher levels of anxiety symptoms and a poorer quality of life in the ‘non-trigger’ group; however, no statistically significant difference was noted between the groups. Clinician rated data noted that eight (26.7%) participants had disimproved since their previous clinical review (prior to COVID-19 restrictions) with 14 (46.7%) participants demonstrating an improvement in their mental health since their most recent review. There was no difference between individuals with a ‘trigger’ and ‘non-trigger’ disorder demonstrated in this regard (Table 2, Fig. 1). Examining individuals with OCD alone compared to other anxiety disorders demonstrated no differential impact of COVID-19 restrictions in relation to anxiety or mood symptoms, or social and occupational functioning or quality of life (Table 3). Only one individual with OCD had a disimproved mental state on clinician scoring compared to seven participants (38.9%) with other anxiety disorders; however, this finding was not statistically significant.

**Table 3.** OCD compared to other anxiety disorders

Variable	OCD (n = 12) n (%)	Other Anxiety Disorders (n = 18) n (%)	Statistics χ^2^, df, p
**CGI-S**			9.97, 5, 0.03*
Not currently illness	0 (0.0)	1 (5.6)	
Borderline illness	4 (33.3)	0 (0.0)	
Mild illness	4 (33.3)	11 (61.1)	
Moderate illness	3 (25.0)	5 (27.8)	
Marked illness	0 (0.00)	1 (5.6)	
Severe illness	1 (8.3)	0 (0.0)	
**Effect on Mental Health**			1.83, 2, 0.44*
Improvement	1 (8.3)	0 (0.0)	
No change	6 (50.0)	8 (44.4)	
Disimprovement	5 (41.7)	10 (55.6)	
**Effect on Anxiety Symptoms**			1.40, 2, 0.57*
Improvement	2 (16.7)	1 (5.6)	
No change	6 (50.0)	8 (44.4)	
Disimprovement	4 (33.3)	9 (50.0)	
**Clinician Rated Impression**			4.26, 2, 0.14*
Improvement	8 (66.7)	6 (33.3)	
No change	3 (25.0)	5 (27.8)	
Disimprovement	1 (8.3)	7 (38.9)	

	Mean (SD)	Mean (SD)	t, p
**Psychometric Data**			
BAI	6.08 (6.75)	16.83 (12.92)	2.64, 0.01
HAM-A	8.42 (5.73)	12.82 (8.89)	1.51, 0.14
GAF	61.58 (14.84)	62.28 (14.84)	0.15, 0.89
**Likert Scales Utilised**			
Anxiety	3.0 (2.7)	4.3 (3.0)	1.23, 0.23
Mood	2.5 (3.0)	3.8 (3.5)	1.09, 0.28
Social functioning	4.6 (2.9)	4.5 (3.1)	0.75, 0.94
Occupational functioning	2.3 (3.2)	3.5 (3.6)	0.91, 0.36
Quality of life	3.8 (2.6)	4.6 (2.7)	0.93, 0.42

CGI-I = Clinical Global Impression Improvement, CGI-S = Clinical Global Impression Severity, BAI = Beck Anxiety Inventory, HAMA-A = Hamilton Anxiety Rating Scale, GAF = Global Assessment of Function, Y-BOCS = Yale-Brown Obsessive Compulsive Scale

* Fisher’s exact test utilised

Clinician Rated Impression = CGI-I modified version

Analyses were repeated with non-parametric analytical techniques (Mann–Whitney U test) as Likert scale data were not normally distributed with similar findings demonstrated.

### Co-morbid psychiatric or physical health disorders

COVID-19 restrictions did not significantly impact individuals with a co-morbid psychiatric disorder to a greater extent than those participants without a co-morbid psychiatric disorder in relation to subjective or objective changes in anxiety or mood symptoms, social and occupational functioning, quality of life or clinician rated impression of mental state (Table 4). Similarly, physical health comorbidity was not associated with a differential impact of COVID-19 restrictions.

**Table 4.** Co-morbid mental health disorders

Variable	Co-morbid Disorder		
	Yes (n = 13) n (%)	No (n = 17) n (%)	Statistics χ^2^, df, p
**COVID-19 Testing**	0 (0.0)	2 (15.4)	2.80, 1, 0.18
**Effect on Mental Health**			2.17, 2,0.25
Improvement	1 (7.7)	0 (0.0)	
No change	7 (53.8)	7 (41.2)	
Disimprovement	5 (38.5)	10 (58.8)	
**Effect on Anxiety Symptoms**			3.925,2,0.175
Improvement	2 (15.4)	1 (5.9)	
No change	8 (61.5)	6 (35.3)	
Disimprovement	3 (23.1)	10 (58.8)	
**Clinician Rated Impression**			4.693,2, 0.28
Improvement	5 (38.5)	9 (52.9)	
No change	6 (46.2)	2 (11.8)	
Disimprovement	2 (15.4)	6 (35.3)	

	Mean (SD)	Mean (SD)	t, p
**Likert Scales Utilised**			
Anxiety	3.2 (3.1)	4.3 (2.8)	1.05
Mood	2.9 (3.4)	3.6 (3.3)	0.54
Social functioning	4.0 (3.2)	4.9 (2.7)	0.86
Occupational functioning	3.1 (4.1)	3.0 (3.0)	0.06
Quality of life	3.9 (2.8)	4.5 (2.6)	0.70
**Psychometric Data**			
BAI	14.9 (16.0)	10.7 (7.8)	0.95
HARS	10.7 (9.1)	11.3 (7.2)	0.97
GAF*	58.1 (13.7)	65.0 (10.0)	1.61
Y-BOCS (n = 12)	14.0 (5.5)	14.67 (7.3)	0.28

* Higher scores indicate less severe illness

## Discussion

To our knowledge, this is the first study to date to examine the impact of COVID-19 and associated mandated restrictions for individuals with pre-existing diagnosed mental disorders who are attending secondary mental health services. Participants reported a deleterious impact of COVID-19 on anxiety symptoms. However, this impact was not marked, with objective ratings by clinicians noting no deterioration for most participants. The greatest impact of the COVID-19 restrictions is related to reduced social functioning and quality of life. The presence of co-morbid psychiatric or physical health difficulties was not associated with additional symptomatology or impairment.

An increase in anxiety symptoms secondary to COVID-19 has been noted in individuals without pre-existing mental disorders. A recent population study in China demonstrated overall prevalence of anxiety and/or depression of 20% (Li *et al*. 2020). Another study found that 30% of individuals noted anxiety symptoms and 17% noted depressive symptoms of at least moderate severity (Wang *et al*. 2020). These figures are consistent with our study where 40% of participants reported a deleterious effect on anxiety symptoms. However, this effect was predominantly mild in severity, and when compared with their last clinical review (prior to COVID-19 restrictions); only 27% of participants demonstrated deterioration in their mental state. It is likely that some of these participants may have demonstrated deterioration in their mental state irrespective of the COVID-19 pandemic. Of note, 47% of participants demonstrated an improvement in their mental state since their most recent pre-COVID-19 restrictions clinical review, which is unlikely to be attributable to restrictions secondary to COVID-19 and is instead likely to be related to the natural course of their illness and its treatment.

Comparisons between individual anxiety disorders are tentative, given the relatively low numbers of study participants. However, individuals whose anxiety symptoms occur predominantly without a trigger, such as in GAD, demonstrated anxiety symptoms and quality of life impairment scores in excess of those who experience anxiety symptoms predominantly due to a triggering effect, albeit these findings were statistically not significant. Individuals with OCD alone appeared to have been less severely impacted by COVID-19 restrictions in relation to anxiety symptoms and quality of life impairment (non-significant), with clinician rating noting only one participant with OCD demonstrating deterioration in symptomatology.

There are a number of putative reasons why individuals with established anxiety disorders in this study have, despite 2 months of restrictions in an active viral pandemic, not demonstrated a significant deterioration in symptomatology. Overall, this cohort of patients attending a community mental health team was stable from a mental health perspective, albeit with some ongoing anxiety symptoms. Participants had been attaining some ongoing supports where appropriate from a community mental health team member(s), and perhaps unlike individuals without a pre-established diagnosis or those individuals experiencing anxiety symptoms *de novo*, participants had an awareness of how to access supports and were aware of anxiety management techniques due to their engagement with mental health services. Second, five patients in this study (16%) had a diagnosis of social phobia or agoraphobia, where anxiety is related to social contact or leaving a perceived place of safety, for example, their home environment. It is interesting that three patients (17.6%) with a diagnosis of agoraphobia, social phobia or OCD reported a subjective improvement in anxiety symptoms, compared to no patients from the other group of diagnoses. In addition, more patients with agoraphobia, social phobia or OCD were deemed improved overall by CGI-I (9 patients, 52.9%) compared to the other anxiety disorders (5 patients, 38.5%), albeit this difference was not statistically significant. It is possible that the reduced social interaction and travel restrictions associated with the COVID-19 pandemic directly impacted positively on the symptomatology of patients with phobic disorders. Third, none of the individuals in this cohort engaged in harmful use of psycho-active substances and although 50% of the cohort consumed alcohol, no participant was engaged in consuming alcohol above the Health Service Executive recommended low-risk alcohol guidelines (https://www2.hse.ie/wellbeing/alcohol/improve-your-health/) Finally, a diagnosis of a mental disorder including an anxiety disorder does not mitigate against an individuals’ ability to be resilient. It is likely in this cohort that many participants are engaged not alone in appropriate coping mechanisms but are also demonstrating significant resilience (‘positive adaptation, or the ability to maintain or regain mental health, despite experiencing adversity’ (Herrman *et al.* 2011)).

There are a number of limitations with this study, the most significant of which were the modest sample size of 30 participants and the absence of a control group. Consequently, caution is required in interpretation of findings between different anxiety disorders. However, to date, no other cross-sectional studies have been conducted in this patient cohort, and this study can serve as a pilot study for future research studies with larger number of participants across a range of anxiety disorders. Second, as this study was undertaken within one community mental health team, it is possible that the findings may not be generalisable to other services with differential resources or co-morbid disorders. Our cohort of patients had a female predominance, which is consistent with existing literature (Kessler *et al.* 2012).

Finally, separating individuals with anxiety disorders into ‘trigger’ and ‘non-trigger’ groups is not a currently recognised diagnostic categorisation. The rationale for such delineation relates to GAD, mixed anxiety disorder and panic disorder being categorised under the same ICD-10 subheading (F41) and symptoms occurring in the absence of an external trigger. Phobic disorders (F40) and OCD (F42) are not categorised under the same ICD-10 sub-heading but symptoms of these disorders frequently relate to an external triggering factor. Given the relatively small cohort of participants, comparative analysis of individual anxiety disorders was not feasible. The terms were deemed appropriate by the authors given the clinical differences between disorders with a defined anxiety-provoking ‘trigger’ and those without. We additionally examined individuals with OCD alone compared to other anxiety disorders, in addition to including individuals with OCD in a ‘trigger’ disorder group, and future studies might potentially be better powered to compare the impact of COVID-19 between individuals with OCD and GAD.

## Conclusion

Two months into the COVID-19 pandemic and its restrictions, the impact on individuals with pre-existing anxiety disorders has been modest, with preliminary evidence demonstrating minimal increases in subjective symptoms of anxiety and reduced social functioning. Future research studies, including qualitative studies, might more clearly delineate the potential adverse sequelae of COVID-19 restrictions on individuals with diagnosed anxiety disorders and ascertain factors associated with positive and negative coping strategies during the COVID-19 pandemic.
